# TORC2 and MAPK signaling pathways regulate mitochondrial degradation induced by iron starvation in *Schizosaccharomyces pombe*

**DOI:** 10.1016/j.jbc.2025.110524

**Published:** 2025-07-25

**Authors:** Rong Li, Jinjie Shang, Ying Huang

**Affiliations:** Jiangsu Key Laboratory for Pathogens and Ecosystems, School of Life Sciences, Nanjing Normal University, Nanjing, China

**Keywords:** mitochondrial degradation, TORC2, Sty1, respiration, calcineurin

## Abstract

Iron is essential for life as it participates in metabolic processes, including DNA synthesis, respiration, and photosynthesis. In this study, we show that iron starvation induced by 2,2′-dithiodipyl (DIP) causes mitochondrial dysfunction, impairs mitochondrial function, including mitochondrial membrane potential (ΔΨ_m_) and respiration, and induces mitochondrial degradation in the vacuole of *Schizosaccharomyces pombe*. The DIP-induced mitochondrial degradation is independent of components of the core autophagy machinery and the ESCRT machinery examined here. We demonstrate that the target of rapamycin complex 2 (TORC2) and its sole target, the AGC kinase Gad8, and the mitogen-activated protein kinase (MAPK) Sty1 play positive roles in regulating iron starvation-induced mitochondrial degradation. The reduction in the level of mitochondrial degradation in Δ*gad8* cells could be restored to wild-type-like levels by treating Δ*gad8* cells with chloramphenicol (CAP) and NaN_3_, two inhibitors of mitochondrial respiration, and by deleting genes encoding components important for mitochondrial electron transport chain (ETC). Disruption of Ca^2+^ signaling through deletion of genes encoding the Ca^2+^ channel proteins Yam8 and Cch1 and the regulatory subunit of calcineurin Cnb1 also restored mitochondrial degradation in Δ*gad8* cells. Our results suggest that the Sty1 MAPK participates with TORC2-Gad8 signaling in regulating DIP-induced mitochondrial degradation. Our results also suggest that TORC2-Gad8 signaling regulates iron starvation-induced mitochondrial degradation through regulation of mitochondrial respiration and Ca^2+^ signaling.

Autophagy is an evolutionarily conserved process in eukaryotes, wherein autophagic cargoes, such as proteins, aggregates, organelles, and pathogens, are transported into lysosomes or vacuoles (yeast lysosomes) *via* double-membrane vesicles called the autophagosome for degradation/recycling (1). Three forms of canonical autophagy have been identified based on molecular mechanisms: macroautophagy, microautophagy, and chaperone-mediated autophagy (CMA) (2). Macroautophagy involves the sequestration of autophagic cargo into the autophagosome, which is then delivered into lysosome/vacuole through membrane fusion between the autophagosome and the lysosome/vacuole. This process can occur selectively or nonselectively (3). In selective macroautophagy, tethering of cargoes to autophagic membranes is mediated by cargo receptors that interact with autophagy-related protein 8 (Atg8) *via* an Atg8-interaction motif (AIM) (4). In contrast, non-selective macroautophagy involves the random engulfment of bulk cytoplasm into autophagosomes, which are then fused to lysosomes/vacuoles for degradation/recycling. Autophagosome formation is initiated in yeast by recruitment of core Atg proteins (also termed core autophagy machinery proteins) at a punctate structure known as the phagophore assembly site (PAS) (5). Among these core Atg proteins, yeast Atg8 and its mammalian ortholog LC3 play a key role in cargo recruitment and autophagosome maturation. Besides, the endosomal sorting complex required for transport (ESCRT) proteins have been reported to facilitate autophagosome closure (6, 7, 8).

Microautophagy is the process by which cytoplasmic materials are directly engulfed by lysosomes/vacuoles (9). Based on molecular mechanisms, microautophagy is classified into two subcategories: the fission type and the fusion type (10). The fission type of microautophagy involves invagination of the lysosomal membrane followed by ESCRT-dependent fission of the lysosomal membrane (10, 11, 12). In this case, the core autophagy machinery is not needed. The fusion type of microautophagy occurs *via* lysosomal membrane invaginations or extensions, which are subsequently sealed by fusion with phagophore-like structures. The formation of these structures relies on the core autophagy machinery and possibly SNARE proteins.

Unlike macroautophagy and microautophagy, which are likely to occur in all eukaryotes, CMA has been observed in mammals but not in yeast (2). CMA does not require membrane reorganization; instead, it relies on the chaperone proteins HSPA8/HSC70 to specifically recognize cytosolic proteins containing the KFERQ motif and direct these proteins to the lysosomal surface for engulfment (13).

Autophagy is regulated by nutrient- and stress-responsive signaling pathways, including the target of rapamycin (TOR) signaling pathway, the cAMP-dependent protein kinase (PKA) pathway, and the mitogen-activated protein kinase (MAPK) pathway (14, 15). TOR is a serine/threonine kinase of the phosphatidylinositol kinase-related kinase (PIKK) family and regulates cellular growth in response to nutritional availability. It exists in two functionally distinct protein complexes: the target of rapamycin complex 1 (TORC1) and complex 2 (TORC2). In *Saccharomyces cerevisiae*, TORC1 inhibits autophagy *via* direct hyperphosphorylation of Atg13, a subunit of the Atg1 complex that is essential for autophagy initiation (16). Hyperphosphorylation of Atg13 by TORC1 reduces its affinity for Atg1 and presumably blocks assembly of the autophagy initiation complex. In contrast, TORC2 and its downstream effector, protein kinase Ypk1, promote autophagy and the general amino acid control (GAAC) response upon amino acid starvation by inhibiting the activity of calcineurin (17). Calcineurin is a calcium/calmodulin-dependent phosphatase central to Ca^2+^ signaling, and functions as a negative regulator of the GAAC response and autophagy (17). In *Schizosaccharomyces pombe*, TORC2 positively regulates TORC1 activity, and the two TORCs function synergistically to regulate autophagy under nutrient starvation (18).

The PKA pathway plays an essential role in cell growth, differentiation, and homeostasis. In *S. cerevisiae* and mammals, PKA activity negatively regulates autophagy through targeting Atg1/ULK1 (19). In *S. pombe*, the PKA pathway represses glucose-starvation-induced autophagy by downregulation of transcription factor Rst2, which activates the expression of genes required for autophagy induction under limited glucose availability (20).

The MAPK pathway plays a critical role in the adaptation of eukaryotic cells to different environmental conditions (21). In *S. pombe*, the Sty1 MAPK pathway controls the response of the cells to multiple environmental stresses, such as heat, osmotic stress, oxidative stress, nutrient limitation, and heavy metal toxicity (22, 23, 24, 25). It has been discovered that the Sty1 MAPK pathway promotes autophagy in response to nutrient depletion, such as nitrogen, glucose, sulfur, and phosphorus (18). Another study shows that MAPK Sty1 and its downstream effector Atf1 positively regulate autophagy upon glucose starvation through phosphorylation of Rst2 at S292 (20). In *S. cerevisiae*, the two MAPKs, Slt2 and Hog1, control mitophagy under nitrogen starvation (26).

Mitochondria are eukaryotic organelles involved in many essential cellular processes, including energy production, numerous metabolic pathways, apoptosis, and cell signaling. Thus, maintaining a healthy mitochondrial pool is crucial. Mitochondrial degradation pathways play an important role in maintaining mitochondrial quality by eliminating damaged mitochondria. These degradation pathways include mitophagy and mitophagy-independent pathways (27).

Mitophagy is a selective form of macroautophagy that removes superfluous or damaged mitochondria. It is mediated by ubiquitin-dependent or receptor-dependent mechanisms (28, 29). Ubiquitin-mediated mitophagy relies on ubiquitin chains generated by E3 ubiquitin ligases, which serve as signals for mitophagy (28, 29). The most well-characterized pathway in mammals is the PINK1/Parkin pathway. When mitochondrial insults lead to a loss of mitochondrial membrane potential (ΔΨ_m_), the kinase PINK1 accumulates in the mitochondrial outer membrane (MOM) and phosphorylates the E3 ubiquitin ligase Parkin. These result in the recruitment of Parkin from the cytosol to mitochondria, where it mediates ubiquitination of MOM proteins. This increased ubiquitination of MOM proteins leads to the recruitment of autophagic adapter proteins and the subsequent recruitment of the mitophagy machinery to initiate mitophagy.

In mammals, autophagy receptors located on the mitochondrial membrane, including BNIP3L (30), BNIP3 (31), FUNDC1 (32), AMBRA1, and PHB2, induce selective mitophagy by binding to LC3 through their LC3-interaction regions (LIRs). Similarly, mitochondria-anchored receptors Atg32 and Atg43 mediate mitophagy by binding to Atg8 through their Atg8-interacting motifs (AIMs) in *S*. *cerevisiae* and *S. pombe*, respectively (33, 34). This binding is essential for tethering Atg8/LC3 to the mitochondria.

Mitochondrial degradation can also be mediated by microautophagy (27). In *S. cerevisiae*, under nonfermentable growth conditions, mitochondrial degradation is mediated by microautophagy that requires Atg proteins and the outer mitochondrial membrane protein Uth1 (35). In mammals, the mitochondrial-derived vesicles (MDVs)-mediated microautophagy plays an important role in mitochondrial turnover under both basal and stress conditions. MDVs are generated by the enclosure of mitochondrial components in mitochondrial membranes, which are subsequently cleaved and delivered to the lysosomes for degradation (36).

Iron is an essential element for all living cells, where it is used in the synthesis of protein cofactors such as iron-sulfur (Fe-S) clusters and heme (37, 38). Some iron-containing proteins are involved in fundamental processes of mitochondria, such as respiration, tricarboxylic acid cycle (TCA), and Fe-S cluster and heme biosynthesis (38, 39). Thus, iron metabolism controls mitochondrial function.

Iron deficiency has been shown to induce mitochondrial degradation. In pathogenic yeast *Candida glabrata*, iron deficiency induces CgATG32-mediated mitophagy (40). In *Caenorhabditis elegans*, mitophagy is induced in response to iron starvation triggered upon frataxin depletion and is a protective mechanism against mitochondrial stress (41). In humans, iron chelator deferiprone (DFP) induces mitophagy, which is mediated by the deSUMOylation enzyme SENP3/mitochondrial fission 1 protein (Fis1), but not PINK1/Parkin. Under iron-deficient conditions, the E3 ubiquitin ligase CHIP is downregulated, leading to the stabilization of SENP3, which mediates deSUMOylation of Fis1. UnSUMOylated Fis1 translocates to mitochondria, where it promotes mitophagy (42, 43).

In this study, we show that 2,2′-dithiodipyl (DIP)-induced mitochondrial degradation in *S. pombe* is independent of components of the core Atg machinery and the ESCRT machinery. We further show that TORC2-Gad8 signaling and Sty1 signaling positively regulate DIP-induced mitochondrial degradation. Finally, we show that mitochondrial dysfunction and Ca^2+^ signaling function downstream of TORC2-Gad8 signaling.

## Results

### Iron deficiency causes growth defects and mitochondrial dysfunction in *S. pombe*

We treated *S. pombe* wild-type (WT) cells with 250 μM DIP, which is known to cause iron deficiency and growth defects (44, 45, 46). Consistent with the previous results, DIP markedly inhibited the growth of *S. pombe* cells on Edinburgh minimal medium (EMM) (Fig. S1*A*). The growth defect caused by DIP could be largely rescued by the supplementation of FeCl_2_ (Fig. S1*A*). *S. pombe* cells were smaller when grown in the presence of DIP (Fig. S1*B*). DIP significantly decreased the number of cells containing the septum, compared to those grown in EMM (Fig. S1*B*).

**Figure 1:**
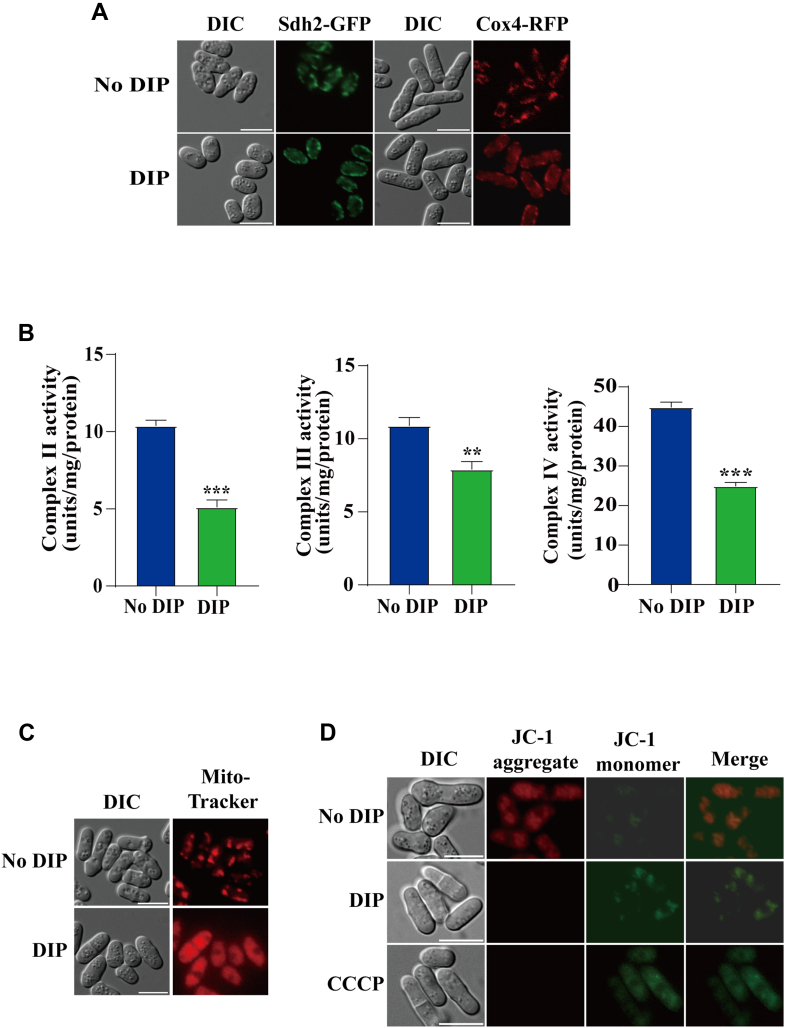
**DIP induces mitochondrial dysfunction in *S. pombe.****A*, cells expressing Sdh2-GFP or Cox4-RFP were grown in minimal medium EMM in the absence of DIP or in the presence of 250 μM DIP for 12 h. GFP and RFP signals were detected by fluorescence microscopy. Scale bars: 10 μm. *B*, cells were grown in EMM in the absence or presence of 250 μM DIP for 12 h, and the activities of complex II, complex III, and complex IV were measured using the DCPIP colorimetric assay. The values are presented as the mean ± SD of three independent experiments. The statistical significance of the mean values was determined by Student's *t* test (∗∗*p* < 0.01; and ∗∗∗*p* < 0.001). *C*, cells were grown in EMM in the absence or presence of 250 μM DIP for 12 h, followed by MitoTracker Red staining. The signals were detected using fluorescence microscopy. Scale bars: 10 μm. *D*, cells were grown in EMM media in the absence or presence of 250 μM DIP for 12 h, or in EMM for 12 h, and then treated with 10 μM CCCP. Cells were stained with JC-1. The fluorescence signal was detected *via* a microscope. Scale bars: 10 μm.

Because iron deficiency is linked to mitochondrial dysfunction, we investigated the effects of DIP on mitochondrial function. First, we examined whether DIP affected mitochondrial morphology. For this, we labeled individual mitochondria in living *S. pombe* cells by genomic tagging of mitochondrial succinate dehydrogenase subunit 2 (Sdh2) and cytochrome *c* oxidase subunit 4 (Cox4) with green fluorescent protein (GFP) and red fluorescent protein (RFP), respectively. Microscopic analysis revealed that mitochondria displayed a diffuse pattern under iron-deficient conditions (Fig. 1*A*).

Next, we examined the activities of mitochondrial complexes II, III, and IV of the electron transport chain (ETC) upon iron deficiency. The activities of complexes II, III and IV were decreased by ∼51%, ∼27%, and ∼44%, respectively, when cells were grown in the presence of DIP (Fig. 1*B*). We also measured ΔΨ_m_ upon DIP treatment using MitoTracker Red, a dye whose accumulation in mitochondria is dependent on ΔΨ_m_ (47). We found that the accumulation of MitoTracker Red in mitochondria was significantly reduced upon DIP treatment (Fig. 1*C*). To confirm this, we monitored ΔΨ_m_ using 5,5′,6,6′-tetrachloro-1,1′,3,3′-tetraethyl-imidacarbocyanine (JC-1), which is a fluorescent probe for analyzing ΔΨ_m_ changes in intact cells (47). JC-1 forms red aggregates in mitochondria with high ΔΨ_m_, whereas it exists as green monomers in cytosol with low ΔΨ_m_ (48). We found that cells treated by DIP emit green fluorescence (Fig. 1*D*). This result is similar to that observed with carbonyl cyanide 3-chlorophenylhydrazone (CCCP), which disrupts ETC function and ΔΨ_m_ (49). Taken together, our findings suggest that iron starvation induces mitochondrial dysfunction.

### DIP induces mitochondrial degradation in *S. pombe*

**Figure 2:**
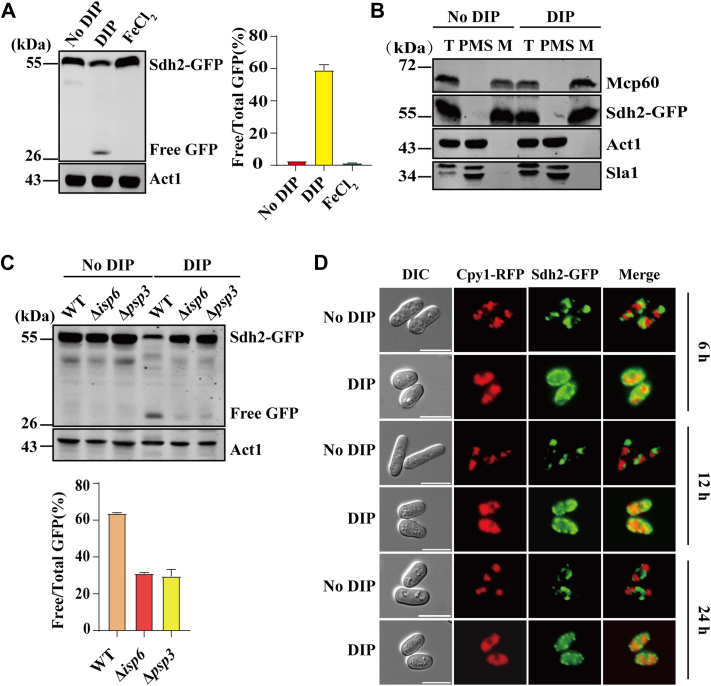
**DIP induces mitochondrial degradation in *S. pombe*.***A*, turnover of Sdh2-GFP. Cells expressing Sdh2-GFP were grown in EMM media in the absence or presence of 350 μM DIP, or EMM media in the presence of 350 μM DIP and 200 μM FeCl_2_ for 12 h. Whole-cell lysates were analyzed by Western blotting using anti-GFP Ab. The ratio of free GFP vs. Sdh2-GFP (mean ± SD) was quantitated from three independent repeats. Act1 serves as a loading control. *B*, analysis of the subcellular localization of Sdh2-GFP. Mitochondria were isolated from cells expressing Sdh2-GFP grown in the absence or presence of 350 μM DIP for 12 h. Whole cells (T), mitochondrial (M), and postmitochondrial supernatant (PMS) were analyzed by Western blotting using anti-HSP6, anti-GFP, anti-Act1, and anti-Sla1 to detect the mitochondrial heat shock protein Mcp60, Sdh2-GFP, actin Act1, and the nuclear protein Sla1, respectively. *C*, Sdh2-GFP processing was decreased in Δ*isp6* and Δ*psp3* cells. WT, Δ*isp6,* and Δ*psp3* cells expressing Sdh2-GFP were grown in EMM medium in the absence or presence of 350 μM DIP for 12 h. Processing of Sdh2-GFP was analyzed as in Fig. 2*A*. *D*, colocation of the mitochondrial marker Sdh2-GFP with the vacuolar marker Cpy1-RFP. Cells expressing Sdh2-GFP and Cpy1-RFP were grown in EMM medium in the absence or presence of 350 μM DIP for 6, 12, and 24 h, and RFP signals were identified with fluorescence microscopy. Scale bars represent 10 μm.

As DIP disrupts mitochondrial function, we next explored whether it could trigger mitochondrial degradation in *S. pombe* cells. In *S. pombe*, the GFP-tagged Sdh2 has been used as a reporter for mitophagy (33, 50, 51). When mitochondria are delivered to the vacuole during autophagy, Sdh2 is degraded, whereas the free GFP accumulates due to its stability in the vacuole. This allows mitochondrial degradation to be monitored through the appearance of free GFP by immunoblotting. To monitor degradation, we constructed a strain expressing GFP-tagged Sdh2 from its endogenous locus. Western blotting showed that while no Sdh2-GFP processing was detected when cells were grown in the absence of DIP, Sdh2-GFP processing was detected when cells were grown in the presence of 350 μM DIP (Fig. 2*A*). We chose this concentration of DIP because it gave the highest level of free GFP (Fig. S2*A*). Furthermore, when cells were grown in the presence of both DIP and FeCl_2_ or FeSO_4_, the processing of Sdh2-GFP was almost completely abolished (Fig. S2*B*), indicating that mitochondrial degradation was induced by iron deficiency. However, the DIP-induced processing of Sdh2-GFP was not abolished by supplementation with FeCl_3_ or Fe_2_(SO4)_3_ (Fig. S2*C*), probably because DIP is a Fe^2+^chelator (52).

We also monitored mitochondrial degradation using the GFP-tagged Atg43 (Atg43-GFP), a mitochondrial outer membrane protein (33). To do so, we constructed an *S. pombe* strain expressing Atg43-GFP under the control of its native promoter. The processing of Atg43-GFP was also observed when cells were treated with DIP (Fig. S2*D*). As iron deficiency impairs mitochondrial function and likely has a negative impact on the import of nuclear-encoded mitochondrial proteins, we determined the localization of Sdh2-GFP upon iron deficiency. For this, we isolated mitochondria from cells expressing Sdh2-GFP under the control of its own promoter, and probed the mitochondrial extracts with antibodies against Sdh2-GFP, Sla1, a nuclear protein essential for tRNA maturation (53), a cytosolic protein Act1, and a mitochondrial protein Mcp60. As shown in Figure 2*B*, Sdh2-GFP was found in the mitochondria-enriched fraction.

We next sought to determine whether the mitochondria were delivered to the vacuole for degradation when cells were treated with DIP. To do so, we first examined whether deletion of *isp6* or *psp3*, which encode vacuole proteases required for autophagy (54, 55), could affect the DIP-induced delivery of mitochondria to the vacuoles, using the Sdh2-GFP processing assay. The results showed that deletion of *isp6* or *psp3* dramatically decreased free GFP levels (Fig. 2*C*). We next examined whether DIP induced delivery of mitochondria to the vacuoles using fluorescence microscopy. To do so, we constructed an *S. pombe* strain expressing GFP-tagged Sdh2 and RFP-tagged vacuolar carboxypeptidase Y (Cpy1) from their respective endogenous loci. Sdh2-GFP and Cpy1-RFP were used as a mitochondrial marker and a vacuole marker, respectively. We observed an overlap of the Sdh2-GFP and Cpy1-RFP signals, suggesting that mitochondrial degradation induced by DIP occurred in the vacuoles (Fig. 2*D*). This overlap of the fluorescence signals disappeared in Δ*psp3* cells, suggesting that *psp3* is required for targeting mitochondria to the vacuole for degradation (Fig. S2*E*). We also observed that DIP treatment resulted in increases in vacuole size and number (Fig. 2*D*).

It is well-known that CCCP induces mitophagy by disrupting ΔΨ_m_ in mammals (56, 57, 58). Because DIP disrupts ΔΨ_m_, we tested whether CCCP could induce mitochondrial degradation in *S. pombe*. The results showed that CCCP did not induce the processing of Sdh2-GFP (Fig. S2*F*). Fluorescence microscopy revealed that Sdh2-GFP signal did not overlap with the Cpy1-RFP signal upon CCCP treatment (Fig. S2*G*). These results revealed that similar to the situation in *S. cerevisiae* (59, 60) but unlike in mammals (56, 57, 58), disruption of ΔΨ_m_ by CCCP does not induce mitochondrial degradation in *S. pombe*, suggesting that CCCP treatment does not induce mitophagy in *S. pombe*.

### DIP-induced mitochondrial degradation in *S. pombe* is independent of core autophagy proteins, autophagic receptor Atg43, and the ESCRT machinery

**Figure 3:**
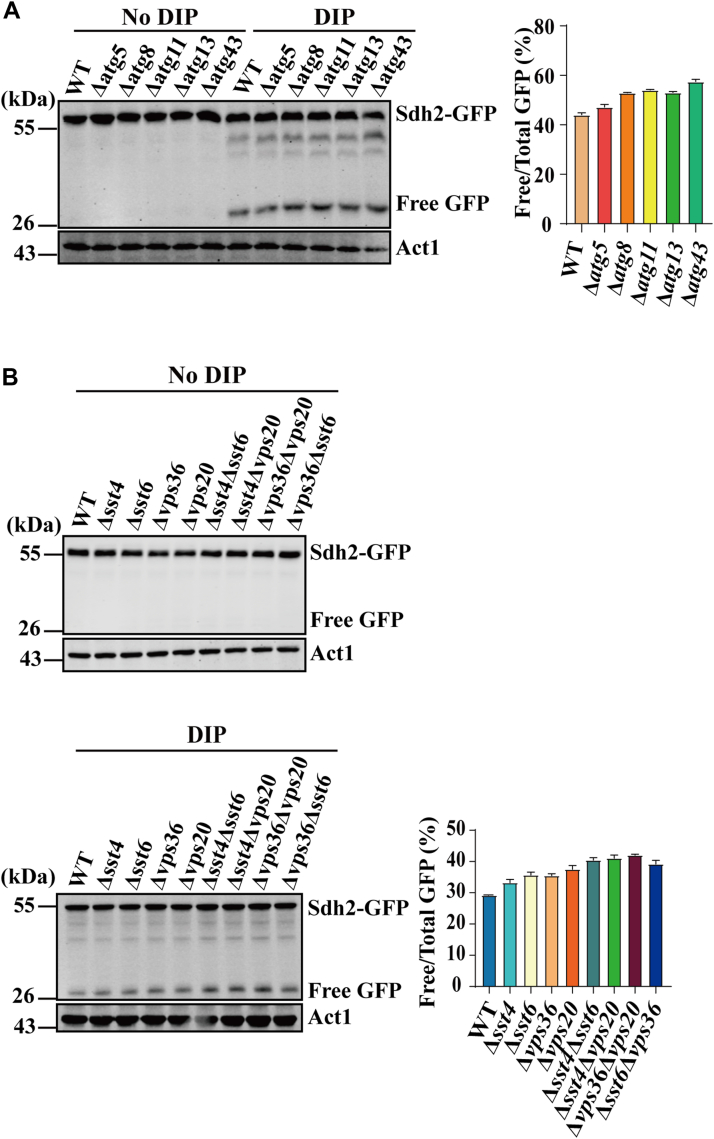
**DIP-induced mitochondrial degradation is independent on the components of core Atg and ESCRT machineries tested here.***A*, deletion of core Atg genes does not affect Sdh2-GFP processing upon iron deficiency. WT, Δ*atg5*, Δ*atg8*, Δ*atg11*, Δ*atg13*, and Δ*atg34* cells expressing Sdh2-GFP were grown in EMM in the absence or presence of 350 μM DIP for 12 h. Processing of Sdh2-GFP was analyzed as in Fig. 2*A*. *B*, deletion of genes encoding ESCRT components does not affect Sdh2-GFP processing upon iron deficiency. Strains WT, Δ*sst4*, Δsst6, Δ*vps36*, Δ*vps20*, Δ*sst4*Δ*sst6*, Δ*sst4*Δ*vps20*, Δ*vps36*Δ*vps20* and Δ*sst6*Δ*vps36* expressing Sdh2-GFP, were grown in EMM in the absence or presence of 350 μM DIP for 12 h. Processing of Sdh2-GFP was analyzed as in Fig. 2*A*.

*S. pombe* contains 18 core Atg proteins required for autophagosome assembly. Among these core Atg proteins, Atg13 is a regulatory subunit of the autophagy initiation complex, the Atg1 kinase complex, which functions downstream of TOR and plays a critical role in autophagy initiation (61). The activation of Atg1 kinase requires Atg11-mediated dimerization (62). The expansion of the PAS (also called phagophore or the isolation membrane) involves the conjugation of a ubiquitin-like protein Atg8 to phosphatidylethanolamine. Atg5 is part of the Atg12-Atg5/Atg16 complex, which is responsible for the lipidation of Atg8 and its recruitment to the PAS (63). We assessed whether the core autophagy proteins Atg5, Atg8, Atg11 and Atg13 are involved in mitochondrial degradation upon iron deficiency. For this, we individually deleted these Atg genes and examined the processing of Sdh2-GFP in each deletion mutant upon iron deficiency. We also deleted the mitophagy receptor Atg43, which targets mitochondria to the autophagy machinery (50). Deletion of these genes did not affect DIP-induced autophagic cleavage of mitochondria as measured by Sdh2-GFP cleavage (Fig. 3*A*).

We next determined whether DIP-induced mitochondrial degradation depends on the ESCRT machinery, which mediates phagophore closure during mitophagy and plays a central role in most microautophagy (64). The ESCRT machinery consists of four major subcomplexes: ESCRT-0, ESCRT-I, ESCRT-II, and ESCRT-III. To examine whether ESCRT mediates DIP-induced mitochondrial degradation, we constructed *S. pombe* single mutants that deleted *sst4* (ESCRT-0), *sst6* (ESCRT-I), *vps36* (ESCRT-II), and *vps20* (ESCRT-III), and double mutants that deleted *sst4* and *sst6*, *sst4* and *sst20*, *sst20* and *sst36*, and *sst6* and *sst36.* As shown in Figure 3*B*, all single and double mutations of ESCRT genes did not have any inhibitory effect on Sdh2-GFP cleavage.

### TORC2 signaling and Sty1 MAPK signaling regulate DIP-induced mitochondrial degradation in *S. pombe*

**Figure 4:**
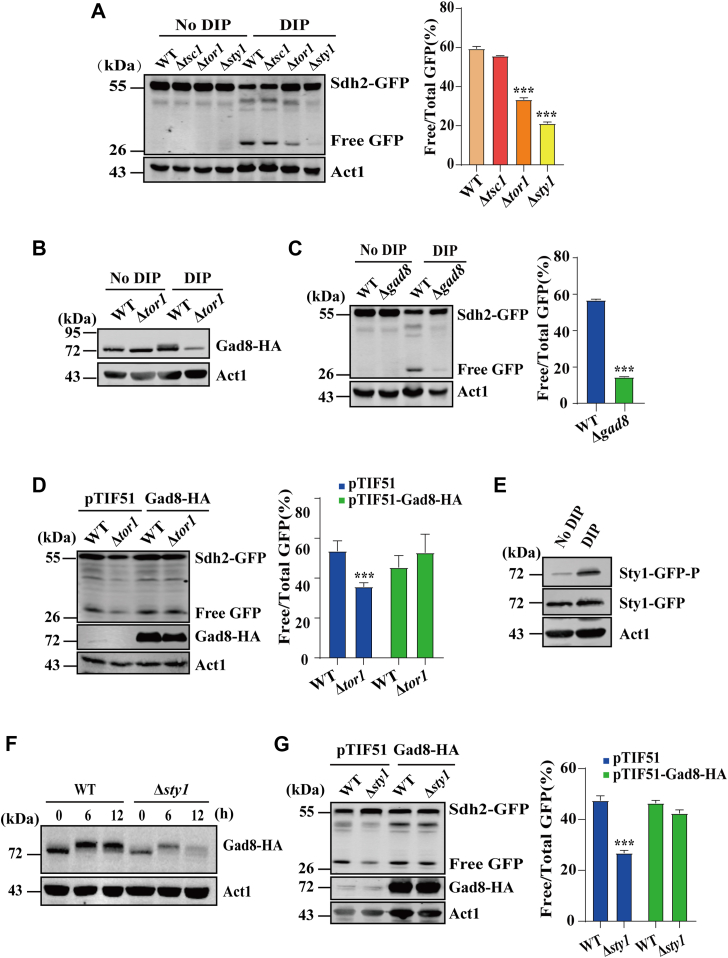
**The TORC2 and MAPK signaling pathways positively regulate autophagy.***A*, deletion of *tor1* and *sty1* reduces the level of Sdh2-GFP processing. WT, Δ*tsc1*, Δ*tor1* and Δ*sty1* cells expressing Sdh2-GFP were grown in EMM in the absence or presence of 350 μM DIP for 12 h. Processing of Sdh2-GFP was analyzed as in Fig. 2*A.* The statistical significance of the mean values was determined by Student's *t* test (∗∗∗*p* < 0.001). *B*, DIP induces Gad8 phosphorylation. WT and Δ*tor1* cells expressing Gad8-HA were grown in EMM in the absence or presence of 350 μM DIP for 12 h. Whole cell lysates were analyzed by Western blotting using anti-HA Ab. *C*, deletion of *gad8* reduces the level of Sdh2-GFP processing. WT and Δ*gad8* cells expressing Sdh2-GFP were grown in EMM in the absence or presence of 350 μM DIP for 12 h. Processing of Sdh2-GFP was analyzed as in Fig. 2*A.* The statistical significance of the mean values was determined by Student's *t* test (∗∗∗*p* < 0.001). *D*, Gad8 overexpression restores the level of DIP-induced mitochondrial degradation in Δ*tor1* cells. WT and Δ*tor1* cells transformed with pTIF51 (empty plasmid) or pTIF51-Gad8-HA and were grown in EMM in the presence of 350 μM DIP for 12 h. Processing of Sdh2-GFP was analyzed as in Fig. 2*A.* The statistical significance of the mean values was determined by Student's *t* test (∗∗∗*p* < 0.001). Whole cell lysates were also analyzed by western blotting with anti-HA Ab to detect Gad8-HA. *E*, DIP induces Sty1 phosphorylation. WT cells expressing Sty1-GFP were grown in EMM in the absence or presence of 350 μM DIP for 12 h. Whole cell lysates were analyzed by Western blotting with antibodies against GFP to detect Sty1-GFP and anti-P-p38 to detect phosphorylation of Sty1 at Thr171 and Tyr173. *F*, deletion of *sty1* reduces Gad8 expression. WT and Δ*sty1* cells expressing Gad8-HA were grown in EMM in the absence or presence of 350 μM DIP for 0, 6, and 12 h. Gad8-HA levels were analyzed by Western blotting with anti-HA Ab. *G*, overexpression of *gad8* in Δ*sty1* cells restores the level of DIP-induced mitochondrial degradation to a level comparable to that of WT cells. WT and Δ*sty1* cells expressing Sdh2-GFP were transformed with pTIF51 or pTIF51-Gad8-HA and were grown in EMM media in the presence of 350 μM DIP. Whole cell lysates were analyzed by Western blotting with anti-GFP and anti-HA Abs. The statistical significance of the mean values was determined by Student's *t* test (∗∗∗*p* < 0.001).

Because the autophagic pathway is tightly controlled by multiple signaling pathways, including TOR signaling and MAPK signaling (18), we investigated the signaling pathways involved in DIP-induced mitochondrial degradation in *S. pombe*. We first examined whether TOR signaling regulates DIP-induced mitochondrial degradation. *S. pombe* has two TORs, Tor2 and Tor1, which are the catalytic subunits of TORC1 and TORC2, respectively. *tor2* is essential whereas *tor1* is non-essential. Since TORC1 negatively regulates autophagy in *S. pombe*, we activated TORC1 by deleting *tsc1*, encoding an upstream negative regulator of TORC1, and analyzed the effect of TORC1 activation on DIP-induced mitochondrial degradation. We found that when *S. pombe* cells were grown in the presence of DIP, the level of free GFP fragments in Δ*tsc1* cells was similar to that in WT cells (Fig. 4*A*). To examine the role of TORC2 in DIP-induced mitochondrial degradation, we inactivated TORC2 by deletion of *tor1*. We found that the level of free GFP fragments was significantly lower in Δ*tor1* cells than in WT cells (Fig. 4*A*). These results suggest that TORC2 but not TORC1 regulates DIP-induced mitochondrial degradation in *S. pombe*.

The serine/threonine protein kinase Gad8 is the sole downstream target of TORC2 in *S. pombe* (65) and is the homolog of *S. cerevisiae* Ypk1 (66). Under conditions of iron deficiency, we observed a mobility shift of the putative phosphorylated form of Gad8 in WT cells. In contrast, a mobility shift of the putative phosphorylated form of Gad8 was absent in Δ*tor1* cells (Fig. 4*B*). We also observed that compared with WT cells, Δ*gad8* cells showed a decrease in the level of free GFP fragments under conditions of iron deficiency (Fig. 4*C*). This reduction could be restored by overexpression of *gad8* (Fig. S3, *A* and *C*). Overexpressing *gad8* in Δ*tor1* cells could restore the level of free GFP fragments to near WT levels (Fig. 4*D*). These findings demonstrate that TORC2-Gad8 signaling plays a crucial role in positively regulating DIP-induced mitochondrial degradation.

We next examined whether Sty1 signaling regulates the DIP-induced mitochondrial degradation. Sty1 is activated through phosphorylation on Thr171 and Tyr173 under a wide variety of stress conditions (67, 68). We first analyzed the phosphorylation of Sty1 under conditions of iron deficiency using anti-phospho-p38 antibody (anti-P-p38) that specifically recognizes Sty1 phosphorylated on Thr171/Tyr173 (69). Western blotting revealed a strong increase in Sty1 phosphorylation under conditions of iron deficiency (Fig. 4*E*). Then, we analyzed the effect of *sty1* deletion on DIP-induced mitochondrial degradation using Sdh2-GFP as a marker. Δ*sty1* cells grown under conditions of iron deficiency exhibited a reduction in the level of free GFP fragments (Fig. 4*A*).

We also examined whether Sty1 has an effect on the TORC2-Gad8 signaling. Western blotting revealed that Gad8 expression levels upon iron deficiency were reduced in Δ*sty1* cells (Fig. 4*F*). Because our study suggested that Gad8 might be the target regulated by Sty1 signaling in response to iron deficiency, we tested whether overexpression of *gad8* could ameliorate impairment of mitochondrial degradation in response to iron deficiency in Δ*sty1* cells. The results showed that ectopic expression of *gad8* in Δ*sty1* cells restored the level of free GFP fragments to near WT levels (Figs. 4*G* and S3). Taken together, these results suggest that Sty1 MAPK signaling crosstalks with TORC2-Gad8 signaling to positively regulate mitochondrial degradation upon iron deficiency.

### Mitochondrial respiration is targeted by TORC2-Gad8 signaling in regulating mitochondrial degradation upon iron deficiency

**Figure 5:**
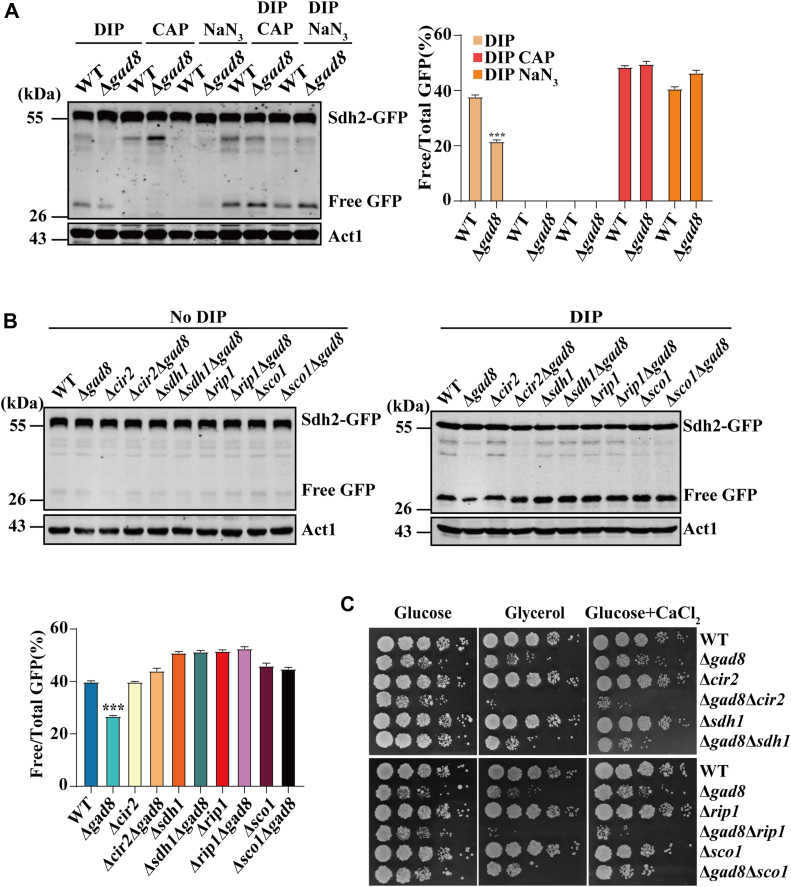
**Mitochondrial respiration functions downstream of TORC2-Gad8 in regulation of DIP-induced mitochondrial degradation.***A*, treatment with CAP and NaN_3_ restores the level of Sdh2-GFP processing to those in the WT cells upon iron deficiency. WT and Δ*gad8* cells expressing Sdh2-GFP, were grown in EMM media in the presence of 350 μM DIP, EMM media containing 2 mg/ml CAP, EMM media containing 5 mM NaN_3_, EMM media containing 350 μM DIP and 2 mg/ml CAP, or EMM media containing 350 μM DIP and 5 mM NaN_3_ for 12 h. Processing of Sdh2-GFP was analyzed as in Fig. 2*A.* The statistical significance of the mean values was determined by Student's *t* test (∗∗∗*p* < 0.001). *B*, deletion of mitochondrial respiration components in Δ*gad8* cells restores the level of DIP-induced mitochondrial degradation to a level comparable to that of WT cells. WT, Δ*cir2*, Δ*gad8*Δ*cir2*, Δ*sdh1*, Δ*gad8*Δ*sdh1*, Δ*rip1*, Δ*gad8*Δ*rip1*, Δ*sco1*, and Δ*gad8*Δ*sco1* cells expressing Sdh2-GFP were grown in EMM media in the absence or presence of 350 μM DIP. Processing of Sdh2-GFP was analyzed as in Fig. 2*A*. The statistical significance of the mean values was determined by Student's *t* test (∗∗∗*p* < 0.001). *C*, deletion of mitochondrial respiration components in Δ*gad8* cells causes severe respiration defects and hypersensitivity to Ca^2+^. WT, Δ*gad8*, Δ*cir2*, Δ*gad8*Δ*cir2*, Δ*sdh1*, Δ*gad8*Δ*sdh1*, Δ*rip1*, Δ*gad8*Δ*rip1*, Δ*sco1*, and Δ*gad8*Δ*sco1* cells expressing Sdh2-GFP were grown in EMM media in the presence of 350 μM DIP for 12 h. The cells were normalized to the same *A*_600_, and 3 μl of serial 10-fold dilutions were spotted on minimal media containing 3% glucose, minimal media containing 3% glycine and 0.1% glucose, or minimal media containing 3% glucose and supplemented with 0.1 M CaCl_2_. The plates were incubated at 30 °C for several days before being photographed.

Previous observations that mitochondrial respiratory function collaborates with TORC2 signaling to regulate autophagy upon amino acid starvation in *S. cerevisiae* (70) prompted us to investigate a possible link between mitochondrial respiratory and TORC2-Gad8 signaling on mitochondrial degradation under iron-depleted conditions. Treatment of WT *S. pombe* cells with chloramphenicol (CAP) and NaN_3_, two well-known inhibitors of mitochondrial respiration (71), did not induce mitochondrial degradation (Fig. 5*A*). Treatment of Δ*gad8* cells with CAP or NaN_3_ was able to restore the level of free GFP fragments to the WT levels under iron starvation conditions (Fig. 5*A*).

To confirm that the rescue of mitochondrial degradation was specifically due to the inhibition of mitochondrial respiration, we performed epistasis studies by deleting individual genes encoding components of mitochondrial respiration complexes including Cir2 (flavoprotein-ubiquinone oxidoreductase), Sdh1 (a subunit of complex II), Rip1 (a subunit of complex III), and Sco1 (a subunit of complex IV) in Δ*gad8* cells. These proteins are important for the assembly of the mitochondrial electron transport chain (71). Deletion of these genes similarly rescued mitochondrial degradation under iron-depleted conditions in Δ*gad8* cells (Fig. 5*B*).

To understand how deletions of individual genes encoding components of mitochondrial respiration increased mitochondrial degradation upon iron deficiency in Δ*gad8* cells, we assessed the growth of the mutant strains on glucose-containing or glycerol-containing minimal medium, which allows only respiratory growth (72). Deletion of *cir2*, *sdh1*, *rip1,* or *sco1* moderately affected cell growth on glycerol-containing medium (Fig. 5*C*). Deletion of *gad8* also moderately affected cell growth on glycerol-containing minimal medium, consistent with its role in promoting mitochondrial respiration. In contrast, Δ*cir2*Δ*gad8*, Δ*sdh1*Δ*gad8*, Δ*rip1*Δ*gad8* and Δ*sco1*Δ*gad8* cells grew much slower than the individual deletion mutants on glycerol-containing medium. As controls, Δ*cir2*Δ*gad8*, Δ*sdh1*Δ*gad8*, Δ*rip*1Δgad8, and Δ*sco1*Δ*gad8* cells displayed a mild slow-growth phenotype when grown on glucose-containing minimal medium, where mitochondrial respiration is largely inhibited (Fig. 5*C*). These results suggest that deletions of individual genes encoding components of mitochondrial respiration in Δ*gad8* cells severely compromised mitochondrial respiration (Fig. 5*C*).

It has been shown that calcineurin activity (see below) is reduced in cells deficient in both mitochondrial respiration and TOCR2-Ypk1 (70). Because *S. pombe* cells deficient in calcineurin activity are hypersensitive to Ca^2+^ (73), we examined the Ca^2+^-sensitivity of *S. pombe* cells deficient in both mitochondrial respiration and TOCR2-Gad8 signaling. Deletion of genes encoding components of mitochondrial respiration in Δ*gad8* cells resulted in hypersensitivity to Ca^2+^ (Fig. 5*C*), suggesting that respiration-deficient Δ*gad8* cells may have decreased calcineurin activity.

### Ca^2+^ signaling acts downstream of TORC2-Gad8 signaling in mitochondrial degradation of mitochondria upon iron deficiency

Because TORC2 signaling promotes the general amino acid control (GAAC) and autophagy by inhibiting the activity of the Ca^2+^-regulated phosphatase calcineurin in *S. cerevisiae* (17), we tested whether calcineurin is also involved in mitochondrial degradation upon iron deficiency in *S. pombe*. In *S. pombe*, the transcriptional response to Ca^2+^ signaling is mediated by the calcineurin-activated transcription factor Prz1, which controls the transcription of numerous target genes involved in evolutionarily conserved and species-specific functions (74).

**Figure 6:**
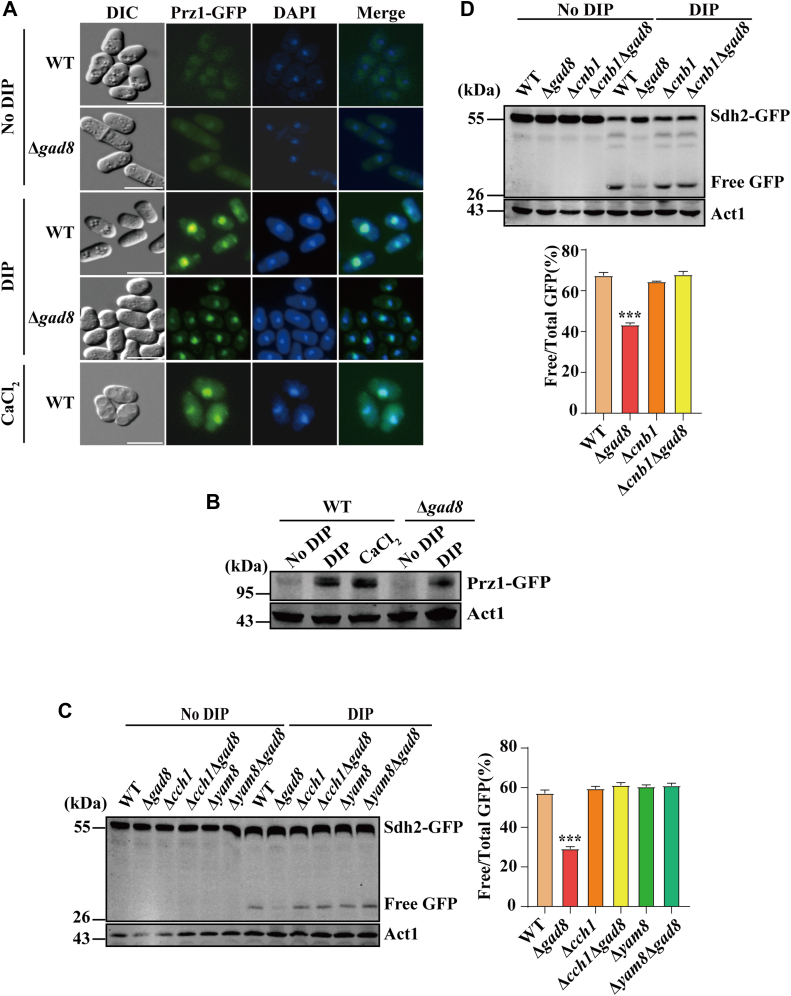
**Ca^2+^ signaling acts downstream of TORC2-Gad8 to regulate DIP-induced mitochondrial degradation.***A*, DIP activates the downstream target of Ca^2+^ signaling. WT and Δ*gad8* cells expressing Prz1-GFP were grown in EMM media in the absence or presence of 350 μM DIP or EMM media supplemented with 0.1 M CaCl_2_ (control) for 12 h. GFP signals were detected by fluorescence microscope and nuclei were detected with DAPI. Scale bars represent 10 μm. *B*, DIP induces Prz1 expression independent of Gad8. WT and Δ*gad8* cells expressing Prz1-GFP were grown in EMM media in the absence or presence of 350 μM DIP or EMM supplemented with 0.1 M CaCl_2_ (for WT). Whole cell lysates were analyzed by Western blotting using anti-GFP Ab. *C*, deletion of *cch1*and *yam8* encoding Ca^2+^ channel proteins in Δ*gad8* cells restores the level of DIP-induced mitochondrial degradation to WT level. WT, Δ*gad8*, Δ*cch1*, Δ*gad8*Δ*cch1*, Δ*yam8*, and Δ*gad8*Δ*yam8* cells expressing Sdh2-GFP were grown in EMM in the absence or presence of 350 μM DIP. Processing of Sdh2-GFP was analyzed as in Fig. 2*A*. The statistical significance of the mean values was determined by Student's *t* test ( ∗∗∗*p* < 0.001) *D*, deletion of *cnb1* encoding the regulatory subunit of *S. pombe* calcineurin in Δ*gad8* cells rescues the processing of Sdh2-GFP upon iron deficiency. WT, Δ*gad8*, Δ*cnb1* and Δ*gad8*Δ*cnb1* cells were grown in EMM in the absence or presence of 350 μM DIP. Processing of Sdh2-GFP was analyzed as in Fig. 2*A*. The statistical significance of the mean values was determined by Student's *t* test (∗∗∗*p* < 0.001).

Prz1 is dephosphorylated and translocated to the nucleus in response to elevated intracellular Ca^2+^ levels (73). Its expression is stimulated by Ca^2+^ in a calcineurin-dependent fashion (73). We first tested whether Prz1 was activated under iron-deficient conditions. We constructed a strain expressing GFP-tagged Prz1 from its endogenous locus. Fluorescence microscopy revealed that Prz1-GFP was accumulated in the nucleus in WT and Δ*gad8* cells in response to iron deficiency (Fig. 6*A*). Western blotting revealed that Prz1 expression in WT and Δ*gad8* cells was upregulated upon DIP treatment (Fig. 6*B*). These results suggest that iron deficiency activates Ca^2+^ signaling irrespective of the presence or the absence of TORC2-Gad8 signaling.

To further investigate the relationship between Ca^2+^ signaling and TORC2-Gad8 signaling in regulating mitochondrial degradation upon iron deficiency, we performed epistatic experiments. The results showed that deletion of genes (*cch1* and *yam8*) encoding Ca^2+^ channel proteins absolutely required for calcineurin activation upon exposure to various stimuli (75), reversed the inhibition of mitochondrial degradation upon iron deficiency caused by deletion of *gad8*, suggesting that Ca^2+^ signaling functions downstream of TORC2-Gad8 signaling in regulating DIP-induced mitochondrial degradation (Fig. 6*C*). Additionally, deletion of *cnb1* encoding the regulatory subunit of *S. pombe* calcineurin had a similar effect (Fig. 6*D*). These results suggest that calcineurin suppresses mitochondrial degradation upon iron deficiency and that TORC2-Gad8 signaling may regulate DIP-induced mitochondrial degradation by repressing the activity of calcineurin.

## Discussion

In this study, we showed that DIP-induced processing of Sdh2-GFP and colocalization of Sdh2-GFP to vacuoles upon iron deficiency in *S. pombe* cells. These results suggest that part of the mitochondria is delivered to the vacuole for degradation upon DIP treatment.

Mitochondrial degradation can be mediated by mitophagy or microautophagy (76). Besides, mitochondria could be removed by noncanonical autophagic pathways, also known as alternative autophagy. For example, mitochondrial clearance in embryonic reticulocytes occurs through Unc-51-like kinase 1 (Ulk1)-dependent Atg5/7-independent macroautophagy and Rab9 appears to be is required for autophagosome is required for autophagosome formation (77). In this study, we found that neither components of the core Atg proteins nor the ESCRT machinery examined here are required for DIP-induced mitochondrial degradation. However, because compensation among the autophagic pathways (78, 79), and many ESCRT proteins have redundant functions (80), the molecular mechanism of DIP-induced mitochondrial degradation remains to be determined.

We also showed that TORC2-Gad8 and Sty1 signaling positively regulate DIP-induced mitochondrial degradation. More importantly, deletion of *sty1* reduced the Gad8 expression levels and overexpression of *gad8* in Δ*sty1* cells increased free GFP fragments from Sdh2-GFP processing. These findings provide evidence that Sty1 signaling and TORC2-Gad8 signaling interact with each other to regulate mitochondrial degradation upon iron deficiency. Interestingly, it has been reported that the Sty1 signaling crosstalks with PKA-Rst2 signaling in the regulation of autophagy upon glucose limitation (20).

We observed that deletion of *gad8* led to a significant decrease in the level of mitochondrial degradation upon iron deficiency based on Sdh2-GFP processing, and that this reduction in the level of mitochondrial degradation in Δ*gad8* cells could be restored to a level resembling that in WT cells by inhibition of mitochondrial respiration in Δ*gad8* cells. These results suggest that mitochondrial respiration may act downstream of TORC2-Gad8 signaling during DIP-induced mitochondrial degradation. We also found that respiratory-deficient Δ*gad8* cells displayed severe mitochondrial respiratory defects, as suggested by severely impaired growth of double mutants defective in mitochondrial respiration and TORC2-Gad8 signaling on glycerol-containing medium. These results suggest that TORC2-Gad8 may regulate mitochondrial respiration. It is interesting to note that the counterpart of TORC2-Gad8 in *S. cerevisiae*, TORC2-Ypk1 is required for normal mitochondrial respiration (81, 82).

The level of mitochondrial degradation in the Δ*gad8* mutant could also be restored by deleting genes encoding components of Ca^2+^ signaling, including Ca^2+^ channel proteins Cch1 and Yam8, and the regulatory subunit of calcineurin Cnb1. These results suggest that Ca^2+^ signaling functions downstream of TORC2-Gad8 signaling. Interestingly, we found that *S. pombe* cells deficient in both mitochondrial respiration and TORC2-Gad8 signaling are hypersensitive to Ca^2+^, a phenotype related to loss of calcineurin activity, suggesting that in respiratory-deficient Δ*gad8* cells may have reduced calcineurin activity. It remains to be determined the functional connections between TORC2-Gad8 signaling, mitochondrial respiratory activity, and calcineurin during DIP-induced mitochondrial degradation in *S. pombe*.

## Experimental procedures

### Strains, media, and growth conditions

*Escherichia coli* DH5α strain was grown in LB medium (1% peptone, 0.5% yeast extract and 1% NaCl) supplemented with or without 50 mg/ml ampicillin at 37 °C. *S. pombe* strains used in this study are listed in Table S1. *S. pombe* cells were propagated in rich YE medium (3% glucose and 0.5% yeast extract) at 30 °C or in Edinburgh minimal medium (EMM) (83). For the Sdh2-GFP processing assay, cells were grown in EMM medium in the absence or presence of the indicated concentration of DIP for 12 h. For assessment of mitochondrial respiratory capacity, cells were grown in EMM medium containing either 3% glucose (fermentative medium) or both 3% glycerol and 0.1% glucose (respiratory medium) for 12 h. For the Ca^2+^ sensitivity assay, cells were grown in EMM medium containing 0.1 M CaCl_2_ for 12h.

Disruption or chromosomal tagging of *S. pombe* genes was made using one-step PCR-based homologous recombination. For gene disruption, the disruption cassette containing ∼500 bp DNA sequence immediately upstream of the target gene' open reading frame (ORF), the selectable marker and ∼500 bp DNA sequence immediately downstream of the target gene' ORF was generated by overlap extension PCR using plasmids pFA6a-kanMX6, pFA6a-hphMX6, or pFA6a-natMX6 and *S. pombe* genomic DNA as templates (83, 84). The disruption cassette was transformed into *S. pombe* cells using a standard LiAc transformation procedure. Correct integration was verified through PCR. For C-terminal tagging of endogenous Sdh2, Atg43, Cpy1, Gad8, Prz1 and Sty1, ∼500 bp immediately upstream and downstream of the stop codon of the corresponding gene in the chromosome were amplified by PCR using the *S. pombe* genomic DNA as a template. A third DNA fragment containing the tag coding sequence, the terminator of *S. cerevisiae* alcohol dehydrogenase 1 gene (*ADH1*), and the antibiotic selectable marker was obtained by PCR using the appropriate cassette as a template. The GFP-hphMX6 cassette from pK18-GFP-hphMX6 (85) was used to introduce the GFP tag at the C-termini of Sdh2, Atg43, Prz1 and Sty1. The 3HA-hphMX6 cassette from pFA6a-HA-hphMX6 (85) was used for the addition of the HA tag to the C terminus of Gad8. The RFP-kanMX6 cassette from pYJ19 (86) was used to fuse the RFP tag to the C terminus of Cpy1. The GFP-natMX6 cassette was also used for addition of the GFP tag at the C termini of Sdh2. The three PCR fragments were then fused using overlap extension PCR and the resulting PCR product was transformed into appropriate *S. pombe* strains.

### Plasmid construction

To construct the plasmid overexpressing HA-tagged Gad8 under the control of the constitutive *tif51* (encoding *S. pombe* translational elongation factor eIF5a) promoter (87), the DNA fragment containing Gad8-HA was obtained by PCR using the genomic DNA of strain yLR24 expressing HA-tagged Gad8 from its endogenous locus as a template. The PCR product was subcloned into pTIF5-kanMX6 (88) using the ClonExpress II One Step Cloning Kit (Vazyme Biotech, C112-01), generating plasmid pTIF51-Gad8-HA.

### Mitochondrial degradation assays in *S. pombe*

*S. pombe* cells expressing Sdh2-GFP or Atg43-GFP from their endogenous loci were cultured overnight in EMM medium and then transferred to EMM medium with or without the indicated concentration of DIP for 12 h. Whole cell lysates were analyzed by Western blotting using anti-GFP Ab (Roche, 11814460001).

### Protein extraction and Western blot analysis

To determine protein levels, whole-cell extracts were prepared *via* alkaline extraction (89). Proteins were separated by 10% or 12% SDS-PAGE and transferred electrophoretically to a nitrocellulose membrane (Cytiva, A29808765). The membrane was first probed with anti-GFP (Roche, 11814460001), anti-HA (Affinity Biosciences, T008), anti-HSP60 (Sangon Biotech, D290707), anti-β-actin (ABclonal, ACO38), anti-Sla1 or anti-P-p38 (Genway, APR00105G), and then probed with IRDye 800CW conjugated goat anti-mouse or anti-rabbit Abs (LI-COR Bioscience). The protein bands on the membranes were detected *via* an Odyssey IR scanner system (LI-COR Biosciences) and quantitated using NIH ImageJ software (imageJ.net/ij), and normalized to Act1. The Western blots shown are representative of at least three independent experiments.

### Fluorescence microscopy

Fluorescence analysis for subcellular localization was performed as previously described (90). Briefly, cells expressing GFP-tagged Sdh2, RFP-tagged Cox4, or RFP-tagged Cpy1 were grown in EMM medium with or without DIP at 30 °C for 12 h. For 4′, 6-diamidino-2-phenylindole (DAPI) staining, cells were harvested, washed with PBS, and then resuspended in 1 μg/ml DAPI solution (Beyotime Biotech, C1002). After incubation for 10 min at 30 °C, cells were harvested and suspended in PBS. All images were obtained on a Zeiss Axio Imager A1 microscope equipped with a PCO Sensicam CCD (charge-coupled-device) camera using excitation/detection parameters of 460 to 480 nm/505 to 530 nm for GFP, 533 to 558 nm/570 to 640 nm for RFP and 335 to 383 nm/420 to 470 nm for DAPI. Data were processed using MetaMorph image processing software (Molecular Devices; https://www.moleculardevices.com).

### Enzyme assays

The activities of mitochondrial complexes II, III, and IV were determined using commercial kits (Solarbio Science and Technology, BC3230, BC3240 and BC0940) according to the manufacturer's instructions. Briefly, cells were grown overnight in EMM medium and diluted to an *A*_600_ of 0.2 and grown for 12 h. Cells were collected and lysed in lysis buffer A (20 mM Tris–HCl, pH 7.4, 15 mM sucrose,1.5 mM EDTA, 0.1% BSA, and 0.2 mM polyvinyl pyrrolidone). The complex II activity was determined by measuring the reduced coenzyme Q-mediated reduction of dichlorophenol-indophenol (DCPIP) using reduced coenzyme Q as substrate with whole cell lysates. The reduction of DCPIP was monitored spectrophotometrically by the loss of absorbance at 605 nm (*ε* = 21 mM^−1^ cm^−1^). The complex III activity was assayed by monitoring the coenzyme Q-mediated reduction of oxidized cytochrome *c* at 550 nm (*ε* = 19.1 mM^−1^ cm^−1^). The complex IV activity was measured by following the oxidation of cytochrome *c*. The activity was calculated from the rate of decrease in absorbance of cytochrome *c* (II) at 550 nm (*ε* = 19.1 mM^−1^ cm^−1^). The activity was expressed as units/mg protein. Protein concentration was determined using the bicinchoninic acid protein assay kit (CWBIO, CW0014S).

### Measurement of the ΔΨ_m_

For MitoTracker Red staining, cells were harvested, washed with PBS, and then incubated for 30 s at 30 °C with 10 μM of MitoTracker Red (Life Technologies, M7512). Cells were harvested and suspended in PBS. The ΔΨ_m_ was determined using an enhanced mitochondrial membrane potential assay kit with a fluorescent dye JC-1 (Beyotime, C2003S) following the manufacturer's protocol. Cells were grown in EMM medium with or without DIP, or EMM medium with 10 μM CCCP for 12 h, and were collected and washed three times in PBS. Then, cells were transferred to JC-1 staining solution and incubated at 37 °C for 20 min. Afterward, cells were washed with JC-1 buffer at room temperature three more times before analysis. Fluorescence signals were detected using a Zeiss Axio Imager A1 microscope (Zeiss) using excitation/detection parameters of 644/665 nm for MitoTracker Red, 514/529 nm for JC-1 monomers and 585/590 nm for J-aggregates. Data were processed using MetaMorph software.

## Data availability

The data that support the findings of this study are available from the corresponding author upon reasonable request.

## Supporting information

This article contains supporting information.

## Conflict of interest

The authors declare that they have no conflicts of interest with the contents of this article.
