# Supplementary material for: TORC2 and MAPK signaling pathways regulate mitochondrial degradation induced by iron starvation in Schizosaccharomyces pombe
Source: J Biol Chem. 2025 Jul 25;301(9):110524. doi: 10.1016/j.jbc.2025.110524 (PMC12446770; doi:10.1016/j.jbc.2025.110524)
Supplement: Supporting Figures [file mmc1.docx]

**Supporting information**

TORC2 and MAPK signaling pathways regulate mitochondrial degradation induced by iron starvation in *Schizosaccharomyces pombe*

Rong Li, Jinjie Shang* and Ying Huang*

Jiangsu Key Laboratory for Pathogens and Ecosystems, School of Life Sciences, Nanjing Normal University, 1 Wenyuan Road, Nanjing 210023, China

Running Title: Iron starvation induces mitochondrial degradation

*Corresponding author

Ying Huang, Ph.D.

Tel: 01186-25-85891263

Fax: 01186-25-85891263

E-mail: [yhuang@njnu.edu.cn](mailto:yhuang@njnu.edu.cn)

*Correspondence may also be addressed to Jingjie Shang.

Email: jinjieshang[@njnu.edu.cn](mailto:fengg@njnu.edu.cn)

Jiangsu Key Laboratory for Pathogens and Ecosystems

School of Life Sciences

Nanjing Normal University

1 Wenyuan Road, Nanjing 210023

China

Contents:

Supporting information Table S1

Supporting information Figure S1

Supporting information Figure S2

Supporting information Figure S3

**Table S1** *S. pombe* strains used in this study

| **Stain** | **Genotype** | **Source** |
| --- | --- | --- |
| 972 | *h^-^* | This lab |
| yLR1 | *h^-^ sdh2::*[*sdh2-GFP-hphMX6*] | This study |
| yLR2 | *h^-^ cox4::*[*cox4-RFP-hphMX6*] | This lab |
| yLR3 | *h^-^ cpy1::*[*cpy1-RFP-hphMX6*] | This study |
| yLR4 | *h^-^ sdh2::*[*sdh2-GFP-hphMX6*] Δ*isp6::kanMX6* | This study |
| yLR5 | *h^-^ sdh2::*[*sdh2-GFP-hphMX6*] Δ*psp3::kanMX6* | This study |
| yLR6 | *h^-^ sdh2::*[*sdh2-GFP-hphMX6*] Δ*atg5::kanMX6* | This study |
| yLR7 | *h^-^ sdh2::*[*sdh2-GFP-hphMX6*] Δ*atg8::kanMX6* | This study |
| yLR8 | *h^-^ sdh2::*[*sdh2-GFP-hphMX6*] Δ*atg11::kanMX6* | This study |
| yLR9 | *h^-^ sdh2::*[*sdh2-GFP-hphMX6*] Δ*atg13::kanMX6* | This study |
| yLR10 | *h^-^ sdh2::*[*sdh2-GFP-hphMX6*] Δ*atg43::kanMX6* | This study |
| yLR11 | *h^-^ sdh2::*[*sdh2-GFP-hphMX6*] Δ*sst4::kanMX6* | This study |
| yLR12 | *h^-^ sdh2::*[*sdh2-GFP-hphMX6*] Δ*sst6::kanMX6* | This study |
| yLR13 | *h^-^ sdh2::*[*sdh2-GFP-hphMX6*] Δ*vps36::kanMX6* | This study |
| yLR14 | *h^-^ sdh2::*[*sdh2-GFP-hphMX6*] Δ*vps20::kanMX6* | This study |
| yLR15 | *h^-^ sdh2::*[*sdh2-GFP-hphMX6*] Δ*sst4::kanMX6* Δ*sst6::natMX6* | This study |
| yLR16 | *h^-^ sdh2::*[*sdh2-GFP-hphMX6*] Δ*sst4::kanMX6* Δ*vps20::natMX6* | This study |
| yLR17 | *h^-^ sdh2::*[*sdh2-GFP-hphMX6*] Δ*vps36::kanMX6* Δ*vps20::natMX6* | This study |
| yLR18 | *h^-^ sdh2::*[*sdh2-GFP-hphMX6*] Δ*vps36::kanMX6* Δ*sst6::natMX6* | This study |
| yLR19 | *h^-^ sdh2::*[*sdh2-GFP-hphMX6*] Δ*tsc1::kanMX6* | This study |
| yLR20 | *h^-^ sdh2::*[*sdh2-GFP-hphMX6*] Δ*tor1::kanMX6* | This study |
| yLR21 | *h^-^ sdh2::*[*sdh2-GFP-hphMX6*] Δ*sty1::kanMX6* | This study |
| yLR22 | *h^-^ sdh2::*[*sdh2-GFP-hphMX6*] Δ*gad8::kanMX6* | This study |
| yLR23 | *h^-^ gad8::*[*gad8-HA-hphMX6*] Δ*tor1::kanMX6* | This study |
| yLR24 | *h^-^ gad8::*[*gad8-HA-hphMX6*] | This study |
| yLR25 | *h^-^* Δ*sty1::kanMX6 gad8::*[*gad8-HA-hphMX6*] | This study |
| yLR26 | *h^-^ sdh2::*[*sdh2-GFP-hphMX6*] Δ*tor1::natMX6* pTIF51*-*gad8*-*HA/kanMX6 | This study |
| yLR27 | *h^-^ sdh2::*[*sdh2-GFP-hphMX6*] pTIF51*-*gad8*-*HA/kanMX6 | This study |
| yLR28 | *h^-^ sty1::*[*sty1-GFP-hphMX6*] |  |
| yLR29 | *h^-^ sdh2::*[*sdh2-GFP-hphMX6*] Δ*sty1::natMX6* pTIF51*-*gad8*-*HA/kanMX6 | This study |
| yLR30 | *h^-^ sdh2::*[*sdh2-GFP-hphMX6*] Δ*cir1*::natMX6 | This study |
| yLR31 | *h^-^ sdh2::*[*sdh2-GFP-hphMX6*] Δ*gad8::kanMX6* Δ*cir1::natMX6* | This study |
| yLR32 | *h^-^ sdh2::*[*sdh2-GFP-hphMX6*] Δ*sdh1::natMX6* | This study |
| yLR33 | *h^-^ sdh2::*[*sdh2-GFP-hphMX6*] Δ*gad8::kanMX6* Δ*sdh1::natMX6* | This study |
| yLR34 | *h^-^ sdh2::*[*sdh2-GFP-hphMX6*] Δ*rip1::natMX6* | This study |
| yLR35 | *h^-^ sdh2::*[*sdh2-GFP-hphMX6*] Δ*gad8::kanMX6* Δ*rip1::natMX6* | This study |
| yLR36 | *h^-^ sdh2::*[*sdh2-GFP-hphMX6*] Δ*sco1::natMX6* | This study |
| yLR37 | *h^-^ sdh2::*[*sdh2-GFP-hphMX6*] Δ*gad8::kanMX6* Δ*sco1::natMX6* | This study |
| yLR38 | *h^-^ prz1::*[*prz1-GFP::hphMX6*] | This study |
| yLR39 | *h^-^ prz1::*[*prz1-GFP::hphMX6*] Δ*gad8::kanMX6* | This study |
| yLR40 | *h^-^ sdh2::*[*sdh2-GFP-hphMX6*] Δ*yam8::kanMX6* | This study |
| yLR41 | *h^-^ sdh2::*[*sdh2-GFP-hphMX6*] Δ*gad8::kanMX6* Δ*yam8::natMX6* | This study |
| yLR42 | *h^-^ sdh2::*[*sdh2-GFP-hphMX6*] Δ*cch1::kanMX6* | This study |
| yLR43 | *h^-^ sdh2::*[*sdh2-GFP-hphMX6*] Δ*gad8::kanMX6* Δ*cch1::natMX6* | This study |
| yLR44 | *h^-^ sdh2::*[*sdh2-GFP-hphMX6*] Δ*cnb1::kanMX6* | This study |
| yLR45 | *h^-^ sdh2::*[*sdh2-GFP-hphMX6*] Δ*gad8::kanMX6* Δ*cnb1::natMX6* | This study |
| yLR46 | *h^-^ atg43::*[*atg43-GFP-hphMX6*] | This study |
| yLR47 | *h^-^ cpy1::*[*cpy1-RFP-hphMX6*] Δ*isp6::kanMX6* |  |
| yLR48 | *h^-^ gad8::*[*gad8-HA-hphMX6*] pTIF51-kanMX6 | This study |
| yLR49 | *h^-^ gad8::*[*gad8-HA-hphMX6*] pTIF51-gad8-HA/kanMX6 | This study |
| yLR50 | *h^-^* Δ*gad8::natMX6* pTIF51-gad8-HA/kanMX6 | This study |
| yLR51 | *h^-^* Δ*sty1::natMX6 gad8::*[*gad8-HA-hphMX6*] pTIF51/kanMX6 | This study |
| yLR52 | *h^-^* Δ*sty1::natMX6 gad8::*[*gad8-HA-hphMX6*] pTIF51-gad8-HA/kanMX6 | This study |
| yLR53 | *h^-^ sdh2::*[*sdh2-GFP-hphMX6*] Δ*gad8::natMX6* pTIF51/kanMX6 | This study |
| yLR54 | *h*^-^ *sdh2::*[sdh2-GFP-hphMX6] Δ*gad8::natMX6*  pTIF51-gad8-HA/kanMX6 | This study |

Fig S1

**
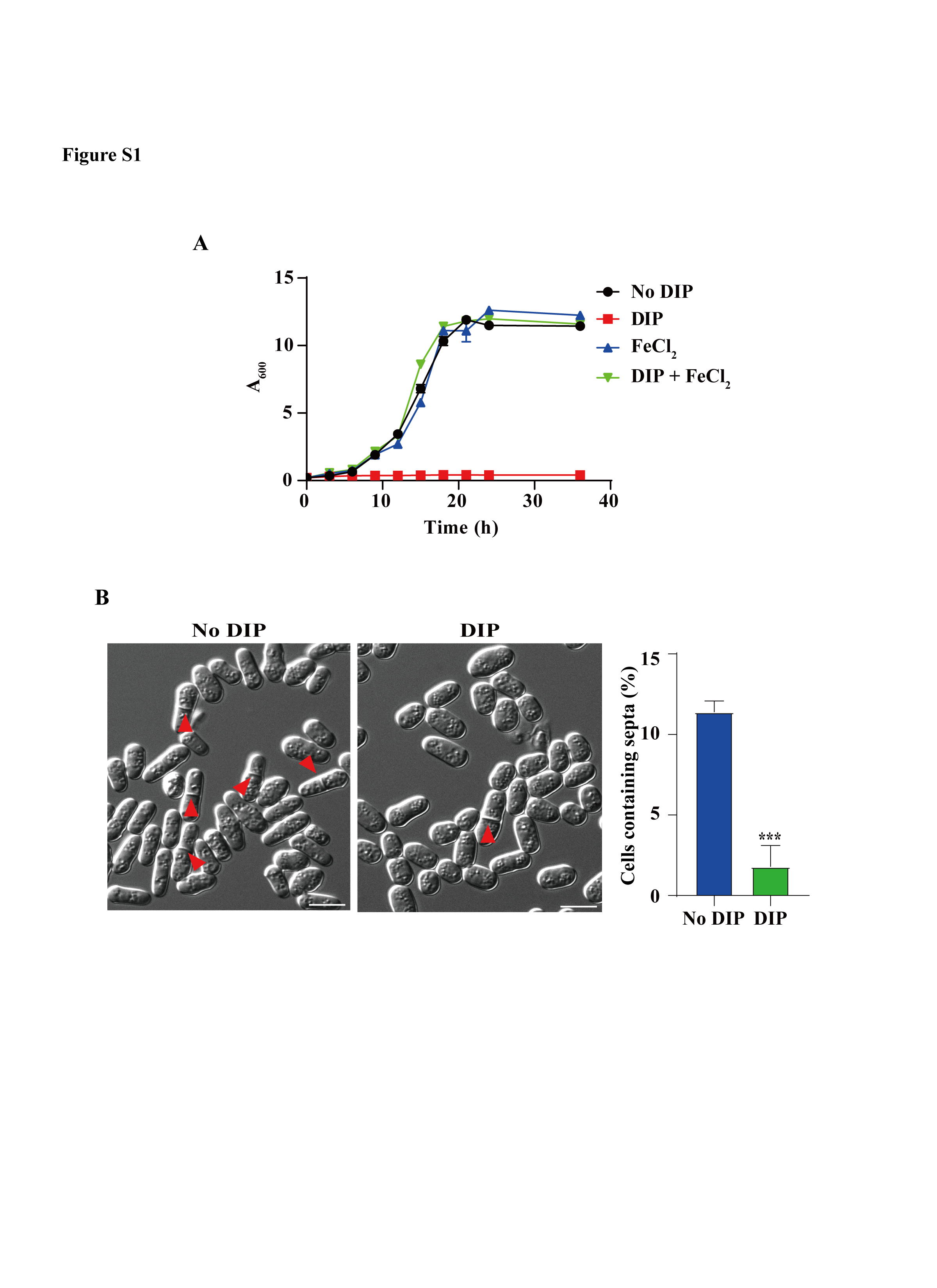
**

**Fig. S1. DIP induces growth defects in *S. pombe*.** *A*, DIP inhibits the growth of *S. pombe* cells. *S. pombe* cells were grown in EMM media with or without 250 μM DIP, EMM with 200 μM FeCl_2_ or EMM with 250 μM DIP and 200 μM FeCl_2_. Cells were collected every 3 h and the *A*_600_ was measured. *B*, DIP significantly decreases the number of cells containing the septum. Cells were grown in EMM with or without 250 μM DIP for 12 h and examined by microscopy. The ratio of cells containing septa vs. total cells (~500) was quantitated from three independent repeats and expressed as mean ± SD (Right). The statistical significance of the mean values was determined by Student's *t* test (^∗∗∗^*p* < 0.001). The red arrow indicates the septum. Scale bars represent 10 μm.

Fig S2


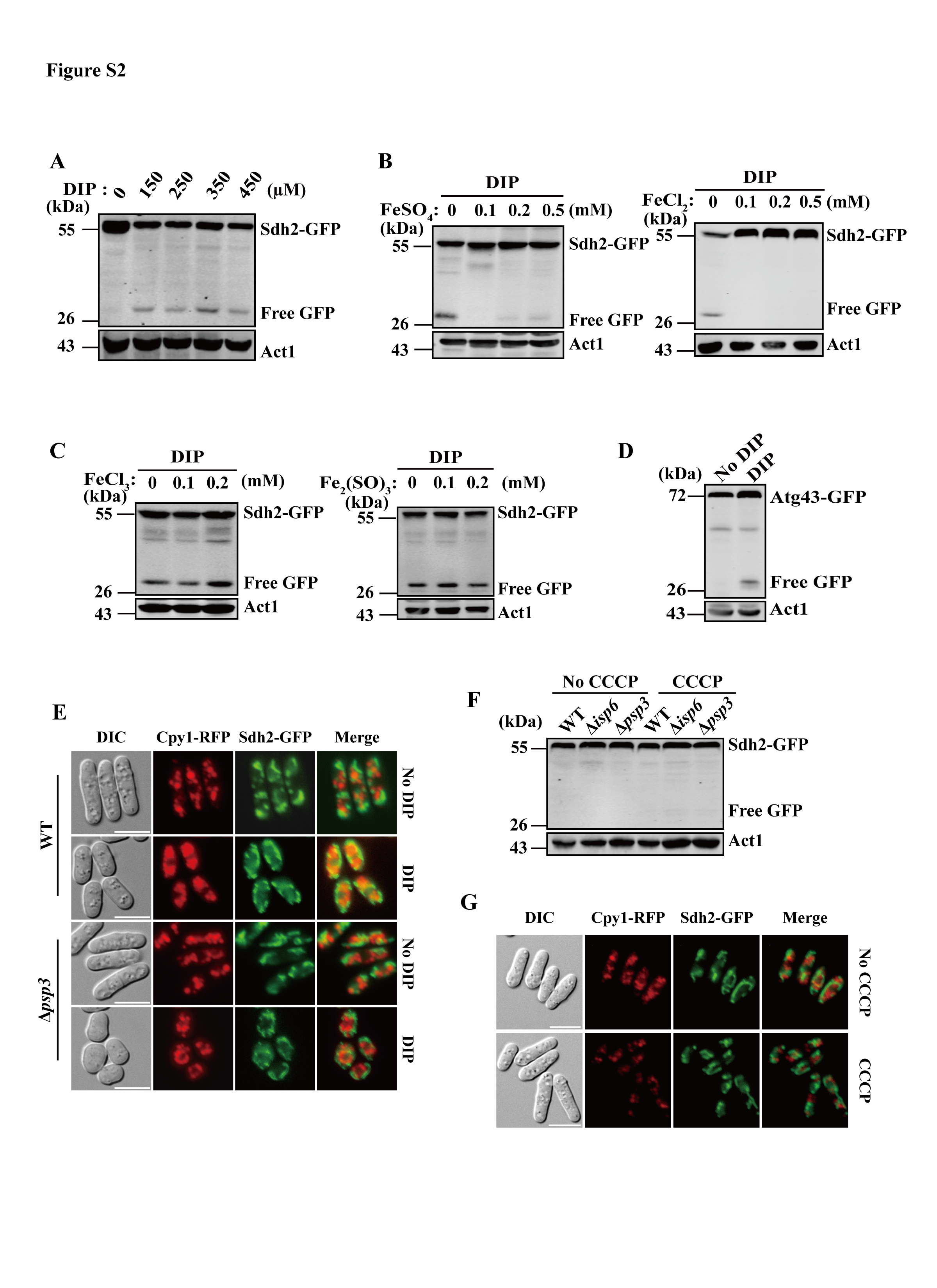


**Fig. S2. Mitochondrial degradation is triggered upon iron starvation.** *A*, processing of Sdh2-GFP in cells treated with varying concentrations of DIP. Cells expressing Sdh2-GFP were grown in EMM media with different concentrations of DIP for 12 h. The processing of Sdh2-GFP was analyzed by Western blotting using anti-GFP Ab. Act1 serves as loading control. *B*, addition of FeSO_4_ or FeCl_2_ restores mitochondrial degradation. Cells expressing Sdh2-GFP were grown in EMM media containing 350 μM DIP and 0, 0.1, 0.2, or 0.5 mM FeSO_4_ or FeCl_2_. The processing of Sdh2-GFP was analyzed by Western blotting as described in (*A*). *C*, addition of Fe_2_(SO_4_)_3_ or FeCl_3_ does not affect mitochondrial degradation. Cells expressing Sdh2-GFP were grown in EMM media containing 350 μM DIP and 0, 0.1 or 0.2 mM Fe_2_(SO_4_)_3_ or FeCl_3_. The processing of Sdh2-GFP was analyzed by Western blotting as described in (*A*). *D*, DIP induces Atg43-GFP degradation. Cells expressing Atg43-GFP were grown in EMM with or without 350 μM DIP. The processing of Atg43-GFP was visualized by Western blotting using anti-GFP Ab. *E*, Colocalization of Sdh2-GFP with the vacuole marker Cpy1-RFP is dependent on the vacuole protease Psp3. WT and Δ*psp3* cells expressing Sdh2-GFP and Cpy1-RFP were grown in EMM with or without 350 μM DIP for 12 h. The cells were then examined by fluorescent microscopy. *F* deletion of *isp6* or *psp3* does not affect Sdh2-GFP processing upon CCCP treatment. WT, Δ*isp6* and Δ*psp3* cells expressing Sdh2-GFP were grown in EMM media in the absence or presence of 10 μM CCCP for 12 h. The processing of Sdh2-GFP was analyzed by Western blotting as described in (*A*). *G*, Fluorescence microscopy revealed that Sdh2-GFP signal did not overlap with the Cpy1-RFP signal upon CCCP treatment. WT cells expressing Sdh2-GFP and Cpy1-RFP were grown in EMM with or without 10 µM CCCP for 12 h. The cells were then examined by fluorescence microscopy.


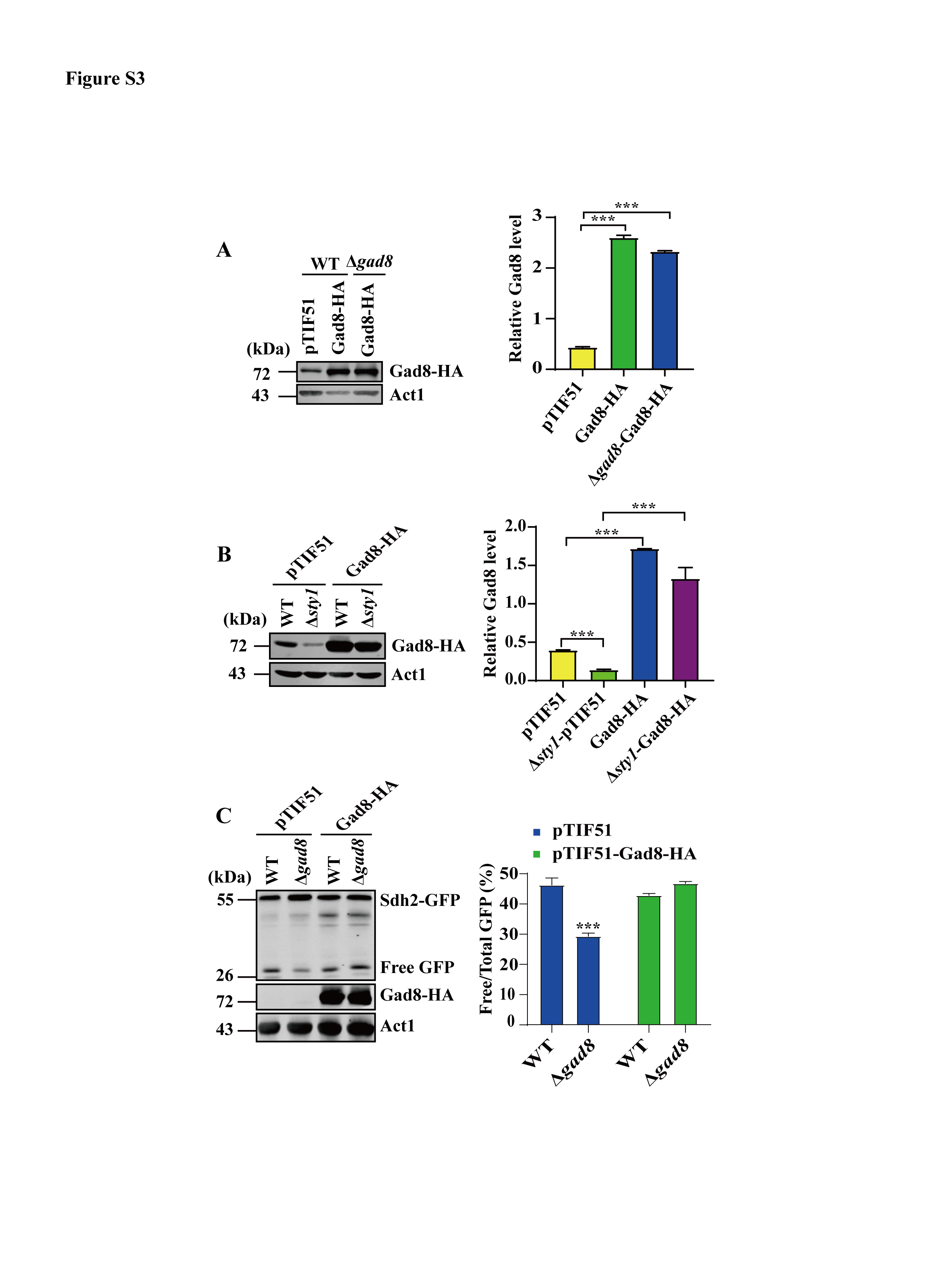


Fig S3

**Fig. S3. Overexpression of *gad8* increases Gad8 levels.** *A*, overexpression of *gad8* in WT and Δ*gad8* cells. WT cells containing pTIF51 (empty vector) and WT cells and Δ*gad8* cells containing pTIF51-Gad8-HA were grown in EMM media with 350 μM DIP for 12 h. Whole cell lysates were analyzed by Western blotting using anti-HA Ab. Gad8-HA levels were quantitated by GraphPad Prism (Right panel). Values represent the mean ± SD of at least three independent experiments. Statistically significant differences were determined by Student's *t* test (^∗∗∗^*p* < 0.001). *B*, overexpression of *gad8* in WT and Δ*sty1* cells. WT and Δ*sty1* cells containing pTIF51 or pTIF51-Gad8-HA were grown in EMM media with 350 μM DIP for 12 h. Whole cell lysates were analyzed by Western blotting as in (*A*). *C*, Overexpression of *gad8* in Δ*gad8* cells restores the degradation of Sdh2-GFP. WT and Δ*gad8* cells containing pTIF51 (empty plasmid) or pTIF51-Gad8-HA were grown in EMM media with 350 μM DIP for 12 h. Whole cell lysates were analyzed by Western blotting using anti-HA, GFP and *β*-actin Abs and the ratio of free GFP vs. Sdh2-GFP (mean ± SD) was quantitated from three independent repeats. Statistically significant differences were determined by Student's *t* test (^∗∗∗^*p* < 0.001).
